# Long-Term Survival in 241 Cases of Intussusception in Cattle and Factors Associated with Mortality

**DOI:** 10.3390/ani14050676

**Published:** 2024-02-21

**Authors:** Laurens Chantillon, Mathilde Laetitia Pas, Lieven Vlaminck, Bart Pardon

**Affiliations:** 1 Department of Internal Medicine, Reproduction and Population Medicine, Ghent University, Salisburylaan 133, 9820 Merelbeke, Belgium; mathilde.pas@ugent.be (M.L.P.); bart.pardon@ugent.be (B.P.); 2 Department of Surgery and Anaesthesiology of Domestic Animals, Ghent University, Salisburylaan 133, 9820 Merelbeke, Belgium; lieven.vlaminck@ugent.be

**Keywords:** bovine, gastrointestinal, risk factors, prognosis, surgery, decision tree, animal welfare

## Abstract

**Simple Summary:**

Intussusception is a frequent cause of mechanical ileus in cattle. However, the prognosis of intussusception is poorly documented and long-term survival, meaning until slaughter or natural death, has never been described. This study aimed to determine survival of cattle surgically treated for intussusception and to identify risk factors associated with mortality. Overall survival was 44.8% until discharge and 39.0% of all animals could complete their life cycle and were eligible for slaughter. Risk factors for mortality were male sex, age < 226 days, heart rate > 95 beats per minute, packed cell volume < 36.5% and time to referral > 4.5 days. The information of the present study can support the decision-making process of farmers and veterinarians to perform surgery or opt for humane euthanasia to the benefit of economics and animal welfare.

**Abstract:**

Intussusception is a frequent cause of mechanical ileus in cattle. Long-term survival has never been described and risk factors for mortality are scarcely documented. A retrospective cohort study on 241 cattle was conducted to determine survival of intussusception and identify risk factors for mortality. Clinical records were matched with the national cattle identification database. Information on possible predictors including clinical examination, ultrasonography, blood-gas analysis and surgery were collected. Survival analysis and decision tree analysis were used. Overall survival was 44.8% until discharge. Of all animals, 39.0% could complete their life cycle and were eligible for slaughter. Male animals and cattle < 226 days old experienced a significantly higher mortality risk (hazard ratio [HR] = 2.1; 95% confidence interval [CI] = 1.4–3.0 and HR = 2.4; 95% CI = 1.7–3.4, respectively). The final model consisted of heart rate (>95 beats per minute) and packed cell volume (<36.5%) with sensitivity and specificity of 60.4% and 49.4%, respectively. A second model consisted of sex (male) and time to referral (>4.5 days) with sensitivity and specificity of 88.0% and 65.6%, respectively. The long-term prognosis for intussusception in cattle appears to be fair. Factors identified in this study may aid in the decision-making process in cases with presumed intussusception to perform the surgery or opt for euthanasia.

## 1. Introduction

Intussusception is among the most frequent causes of mechanical intestinal obstruction leading to ileus and colic in cattle [1,2,3,4,5,6,7]. It occurs in all ages, breeds and sex, but is most common in calves < 2 months old due to the little fat deposition in the mesentery, allowing increased movement of adjacent intestinal segments [5,8,9,10]. Colic signs in cattle are often non-specific, which makes diagnosis challenging. An exploratory laparotomy is often necessary for a definitive diagnosis, but also for treatment, since spontaneous healing with a spasmolytic drug is rare [9,11,12]. The intussusception is most often localized in the small intestines, but caecum and colon can also be intussuscepted, possibly affecting the prognosis [8,13,14].

The prognosis of intussusception is poorly documented. A retrospective multicenter study on 336 cases is available, but only documents postoperative survival (43%) and overall survival rate until discharge (35%) for 57 and 46 animals, respectively [5]. Another retrospective study in 123 cattle showed a slightly higher survival until discharge (44%) [15]. This study presents information concerning further productive life, however, the follow-up was limited to a telephone assessment two years post-discharge and focused on only a subset of the discharged animals. Decision-making in the food animal sector is often driven by economic factors such as the costs of the intervention and estimated future production. Animal welfare also needs to be taken into account, as surgical procedures and the postoperative period may result in prolonged suffering for animals that do not recover. Therefore, the probability of survival until the end of productive life is paramount knowledge for producers in this decision-making process to the benefit of economics and animal welfare. More proficient cattle identification and registration systems will enable us to follow cattle through their life and acquire information about their long-term outcome. These data aid in identifying factors associated with long-term survival in cases of intussusception, as previously seen for necrobacillosis in Belgian Blue cattle [16]. While various factors have been proposed as potential risks for poor surgical outcomes in cases of intussusception, there is currently a lack of available studies on the subject.

Therefore, the objectives of this study were to determine the short- and long-term prognosis (survival), meaning until discharge and until slaughter respectively, of intussusception in cattle and to identify associated risk factors.

## 2. Materials and Methods

### 2.1. Study Design, Data Collection and Selection Procedure

A retrospective cohort study was conducted on the medical records of 248 bovines presented in the Large Animal Internal Medicine clinic, Ghent University. Primary inclusion criteria for the dataset were all cattle diagnosed with intussusception and presented to the clinic between 1 January 2001 and 31 December 2022. Diagnosis was confirmed during surgery, or necropsy for animals in which surgery was not performed. Primary exclusion criteria included presented animals that did not undergo surgery (owners’ decision or pre-operative poor clinical condition) and animals with incomplete or incorrect official ear tag identification (ID). Patient, surgery and hospitalization record data were matched by the ear tag ID with the national cattle identification, registration and movement database (SANITRACE, Animal Health Service Flanders and Federal Agency for the Safety of the Food chain, Torhout/Brussels, Belgium). The date of birth, breed, sex and its final destination (slaughterhouse or destruction facility; i.e., mortality) and the date of this event were retrieved from this database. Secondary exclusion criteria were animals for which no definitive destination could be retrieved. Historical information, clinical examination, blood tests, ultrasonographic examination, surgery records and hospitalization records were used to complete the dataset.

Anamnesis and clinical, ultrasonographic and hematological information were obtained by different veterinarians working in the university clinic, using the same protocols during examinations. Regarding clinical examination, the following parameters were included in the dataset: heart rate (beats per minute (bpm)), respiratory rate (breaths/minute), rectal temperature (°C), skin turgor (sec), color of the mucous membranes (pale/normal/hyperemia), capillary filling time (sec), lumbar reflex, decubitus, increased abdominal tension or distention, feces (presence and abnormal findings e.g., melena), rectal palpation (presence of dilated intestines or intussusception), auscultation and percussion and swinging auscultation of the abdomen (borborygmi, sloshing and steel band sounds).

Concerning laboratory blood variables; pH, pCO_2_ (mmHg), base excess (mmol/L), HCO_3_^−^ (mmol/L), packed cell volume (PCV) (%), L-lactate (mmol/L), sodium (mmol/L), potassium (mmol/L), ionized calcium (mmol/L), chloride (mmol/L) and glucose (mg/dL) were included. These blood results were chosen as they were part of the routine diagnostics conducted upon the animals’ admission to the clinic. All blood samples were taken from the jugular vein or the ventral coccygeal vein with a vacutainer system or needle and syringe. Blood-gas analysis was performed on heparin blood tubes and analyses with RAPIDPoint^®^ 405/500 (Siemens Healthcare, Beersel, Belgium), without temperature correction. Centrifugation was performed to determine PCV. Information concerning ultrasonographic findings (Vivid 7, GE Healthcare, Diegem, Belgium; Esaote, MyLab30 Gold unit, Genua, Italy; Sonosite M-Turbo, Fujifilm Sonosite, Washington, DC, USA) of the abdomen using alcohol 70% as transducer agent were also included; motility and size of jejunal intestines (diameter of ≥4 cm in adult cattle and ≥2 cm in a calf were considered dilated based on personal experience and literature) [17,18,19], presence of bulls-eye sign and free abdominal fluid were recorded. In young animals, a linear probe (5–10 MHz) was used; a sector probe (1–5 MHz) was preferred for adults. Surgery records from the department of Large Animal Surgery and Anesthesia were included about the length and location of the intussusception and presence of free fluid, fibrin or necrosis. Depending on the estimated prognosis by the surgeon and clinician, manual reduction through massage or end-to-end anastomosis was performed. If the intestinal wall was severely damaged or perforated, or if profuse peritonitis was present and the clinical condition of the animal was poor, a negative recommendation was given to the owner. Postoperative hospitalization records included postoperative treatment and euthanasia.

### 2.2. Statistical Analysis

Data were collected in an Excel worksheet (Excel version 2401, Microsoft Inc., Washington, DC, USA) and transferred to SPSS (Statistical Package for the Social Sciences, IBM SPSS Statistics 27.0, Armonk, New York, NY, USA) for statistical analysis. Descriptive statistics, including mean, standard deviation, median and range, were determined using the explore function. To identify factors associated with mortality/survival in cattle undergoing surgery for intussusception, a Cox proportional hazards model was built. The dependent variable was mortality (whether the animal was still alive or slaughtered, or had died via euthanasia or spontaneously). The time between surgery and occurrence of slaughter or mortality was defined as survival time and mortality as the event. Right censoring was applied to animals that had not experienced mortality before the day of slaughter on 5 December 2023, marking the end of the observation period. The odds ratio (OR) for euthanasia during surgery was calculated using logistic regression. Annual mortality risk was determined by the annual number of cattle that did not reach slaughter and the total number of cases each year. Continuous variables, such as respiratory or heart rate and temperature, were tested continuously as well as categorically using reference values. A receiver operating characteristic (ROC)-curve analysis was performed to determine an optimal cutoff point based on the Youden’s index.

All predictors, retrieved from the different records, were first tested univariably for significance and those factors with *p*-value < 0.2 were selected for multivariable modeling. Only the most significant variable was incorporated if Pearson’s and Spearman’s rho correlations were >0.6 for continuous variables. For categorical factors, associations were tested by chi-square testing. Confounding was checked by exploring the associations between the different predictors using univariable logistic regression and introducing associated factors into the multivariable model to check model stability. To build the final multivariable model, a manual backward selection process was used until all remaining variables in the model had a *p*-value < 0.05. Survival function variables of the multivariable model were developed and predictions >50% for mortality were considered as mortality. From this information sensitivity, specificity and accuracy were calculated by means of crosstabs. Kaplan–Meier survival curves were used to visualize the relationship of significant parameters with mortality. Visual inspection of the log minus log plots and construction of time-varying covariates were used to evaluate the proportional hazard assumption.

Next to survival analysis, a decision tree was built based on classification and regression tree analysis. Classification and a regression tree were used as growing methods for the decision tree. Methods and settings were the following: the minimum of decrease in impurity was set at 0.0001 (measured by the Gini index), the growth limit was 15 and 5 observations in the parent node and child node, respectively (maximum depth decision tree determined automatically); number of surrogates was specified as 0. Pruning was automatically performed to avoid additional bias in the model. Missing values were treated as missing values. Diagnostic accuracy of the obtained decision tree was verified by total accuracy, sensitivity and specificity. Finally, the applicability of the used predictors and the practical feasibility of the decision trees were evaluated. Given the limited sample size, the decision tree could only be validated by means of cross-validation.

## 3. Results

### 3.1. Animals

**Figure 1:**
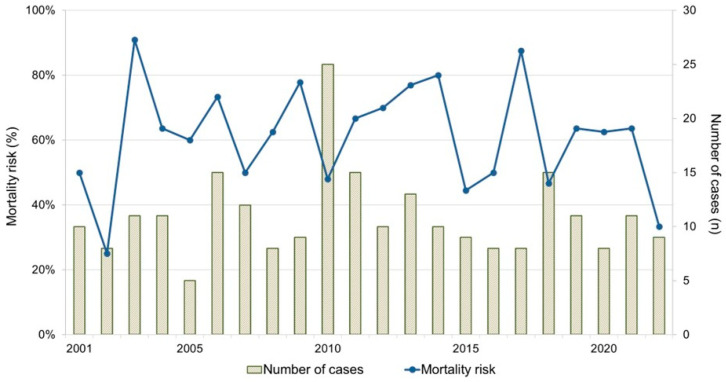
Annual number of cases and mortality risk of cattle with intussusception, presented in the university teaching hospital between 2001 and 2022 (241 cases, Belgium).

A total of 248 bovines that presented to the clinic in the 22-year study period (2001–2022) met the primary inclusion criteria. Of these animals, 241 underwent surgery and could be matched with the national database. In four animals no surgery was performed, and three animals could not be matched with the national cattle database, or a final destination could not be retrieved. Of the included animals, 88.8% (214/241) were Belgian Blue beef cattle, 7.9% (19/241) were Holstein Friesian dairy cattle and 3.3% (8/241) were other breeds such as Blonde d’Aquitaine (*n* = 6), Limousin (*n* = 1) and Maine-Anjou (*n* = 1). Males and females accounted for 18.3% (44/241) and 81.7% (197/241) of the study population, respectively. Mean ± standard deviation (SD) and median (min.–max.) age upon admission to the clinic was 692.9 ± 720.2 and 520.0 (4–4239) days, with 142.0 ± 169.7 and 53 (6–637) for male and 816.0 ± 738.6 and 669.0 (4–4239) for female cattle. Mean ± SD and median (min.–max.) body weight, determined on 154 animals, was 361.3 ± 249.8 and 350.0 (42–890) kg. In Figure 1, the annual case load and mortality risk are shown, displaying a total of 5–15 cases each year (except 2010 with 25 cases) and a yearly mortality risk varying from 25% to 91%.

In 98 cases, colic signs such as teeth grinding, staggered position, lateral decubitus, kicking at abdomen or restlessness were reported. Before being admitted to the clinic, behavioral changes were noticed on average for 2.3 ± 2.1 and 2.0 (0–14) days. Mean ± SD and median (min.–max.) heart rate, respiratory rate and rectal temperature at admission were 88.7 ± 26.3 and 88.0 (29–180) bpm, 39.7 ± 16.5 and 36.0 (12–88) breaths/min and 38.6 ± 0.72 and 38.6 (34.1–40.4) °C, respectively. Table 1 describes the mean ± SD, median, min.–max. and number of cases of each blood-gas parameter included in the dataset. More descriptives for continuous and categorical predictors of mortality are given in Table 2 and Table 3, respectively.

Based on 63 observations, explorative laparotomy showed an average intussusception length of 61.0 cm, with a SD ± 52.2, median of 50.0 and min.–max. of 0.5–300.0 cm. The longest intussusception in discharged cattle measured 150 cm. This particular animal was 616 days old upon entering the clinic and was slaughtered 1676 days post-surgery. Most intussusceptions (74.5%; 114/153) were located in the small intestine and only in 25.5% of the cases in the large intestine. Information about the precise localization of the intussusception was not available.

### 3.2. Mortality

Of the 241 cases, 34.0% (82/241) were slaughtered, 61.0% (147/241) died and 5.0% (12/241) were still alive at the time of analysis. The overall long-term survival rate was 39.0%. Survival times and age of mortality of each group can be found in Table 4. Due to bad clinical condition, severely affected intestines or extensive peritonitis, 29.7% (71/239, information about two cases is missing) of the animals were euthanized during surgery. Mortality mainly occurred within the first two days post-surgery, which accounted for 68.7% (101/147) of total deaths. The majority (82/101) of these cases already died on the day of surgery, of which 71 were euthanized during surgery. Of all animals, 7.9% (19/241) were euthanized postoperatively by the owners’ decision on veterinary advice due to deteriorating clinical condition. Complications resulted in the spontaneous death of 43 animals during hospitalization.

A total of 108 presented animals (44.8%), 7 males and 101 females, were dismissed from the clinic after full postoperative recovery. Of the dismissed animals, 13.0% (14/108), 2 males and 12 females, did not reach slaughter and died prematurely. Detailed information on the cause of death was not available. Survival times and age of mortality of each group can be found in Table 4. Of all animals with small intestinal intussusception, 46.5% (53/114) reached slaughter, in contrast to 15.4% (6/39) with large intestine intussusception. Euthanasia during surgery was performed in 24.6% (28/114) of cases of small intestine intussusception in contrast to 65.4% (22/39) in large intestine intussusception. The *p*-value (<0.001) was significant and OR (95% CI) for euthanasia during surgery was 2.2 (1.4–3.4) for large intestine intussusception in comparison to small intestine intussusception. Post-surgery, no significant difference was seen in long-term prognosis for cases of small and large intestine intussusception.

### 3.3. Multivariable Model and Decision Tree

**Figure 2:**
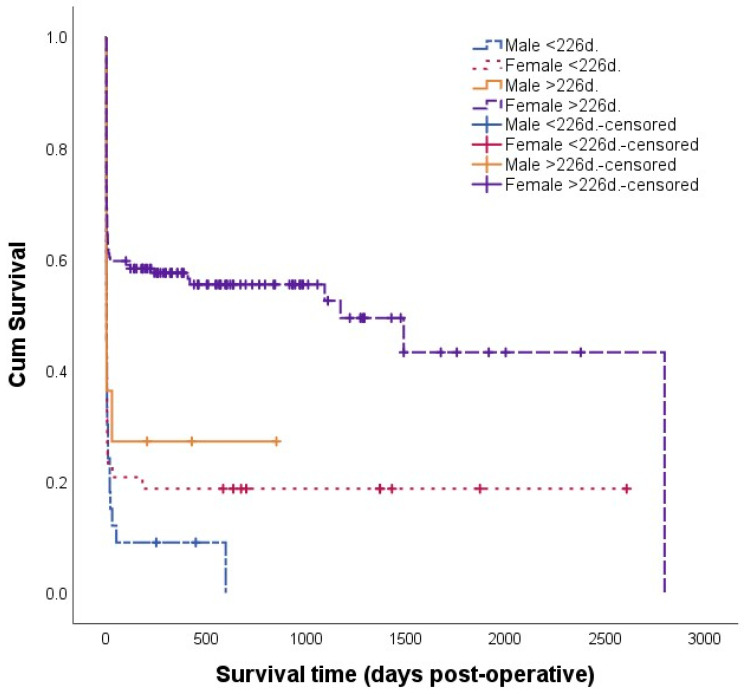
Survival graph for cattle that underwent surgery for intussusception. Cattle were categorized as male < 226 days (d.) old (*n* = 33), female < 226 d. old (*n* = 48), male > 226 d. old (*n* = 11) and female > 226 d. old (*n* = 149). Survival times for females > 226 d. old were significantly (*p* < 0.001) different than those of males < 226 d. old (HR = 2.9; 95% CI 1.9–4.4) and females < 226 d. old (HR = 2.4; 95% CI 1.6–3.5) and significantly (*p* < 0.05) different to those of males >226 d. old (HR = 2.1; 95% CI 1.0–4.4). Survival time for males < 226 d. old, females < 226 d. old and males > 226 d. old were not significantly different from each other (241 cases; 2001–2022; Belgium). Abbreviations: d., days.

The following variables met the inclusion criteria (*p*-value < 0.20) for the multivariable model: sex, age at entry, body weight at entry, time to referral, steroidal anti-inflammatory drugs (SAID) administration before admission, combination non-steroidal anti-inflammatory drugs (NSAID) and SAID administration before admission, antibiotic administration before admission, heart rate, respiratory rate, rectal temperature, pale mucosa color, distended or tensed abdomen, pH, pCO_2_, HCO_3_^−^, PCV, potassium, glucose, L-lactate and location and length of the intussusception. Because combined NSAID and SAID administration before admission, L-lactate and glucose concentration, were categorical variables with less than 20 observations in one group, these were not included in the multivariable model building procedure. Location and length of the intussusception were removed from the multivariable model as these were solely observed during surgery and could not serve as predictive factors. Additionally, distended or tensed abdomen was excluded from the multivariable model building due to its highly subjective nature. ROC analysis identified the optimal cutoff points for some continuous variables, which were more significant in univariable analysis and thereby preferred for the multivariable model. This was the case for age (226 days old), weight (305.5 kg), time to referral (4.5 days), heart rate (95 bpm), respiratory rate (58 breaths/minute), pH (7.415), pCO_2_ (44.95 mmHg), HCO_3_^−^ (22.1 mmHg), PCV (36.5%) and potassium (4.645 mmol/L). Pearson’s and Spearman’s rho analysis showed no significant correlations >0.6 between the continuous parameter temperature and other continuous parameters. However, chi-square analysis showed significant (*p* < 0.05) associations; age and sex especially showed multiple correlations with other parameters. A Kaplan–Meier survival curve in Figure 2 with the parameters of age and sex shows that survival times in females >226 days were significantly higher than those of the other groups.

**Figure 3:**
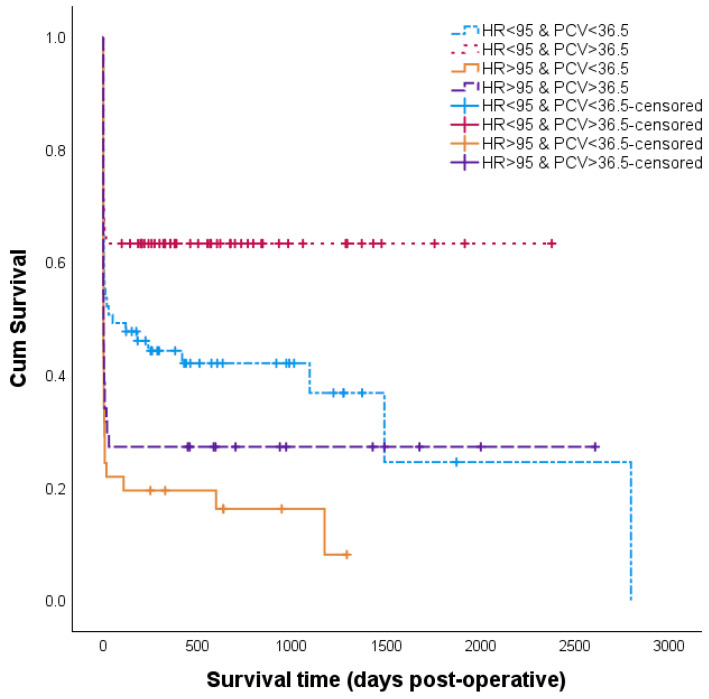
Survival graph of cattle that underwent surgery for intussusception. Cattle were categorized as having heart rate (HR) < 95 bpm and packed cell volume (PCV) < 36.5% (*n* = 67), heart rate < 95 bpm and PCV > 36.5% (*n* = 71), heart rate > 95 bpm and PCV < 36.5% (*n* = 41) and heart rate > 95 bpm and PCV > 36.5% (*n* = 44) (223 cases; 2001–2022; Belgium). Abbreviations: HR, heart rate; PCV, packed cell volume.

Two multivariable models could be built. The first model was formed with male sex and time to referral > 4.5 days. As sex was significantly associated with multiple parameters (container variable) and time to referral is highly dependent on the farmer and less objective, an attempt was made to make a second model holding more explanatory factors, thereby excluding sex and time to referral from the procedure. The second analysis resulted in a model including a heart rate of >95 bpm and a PCV below 36.5%, of which a Kaplan–Meier curve is shown in Figure 3.

**Figure 4:**
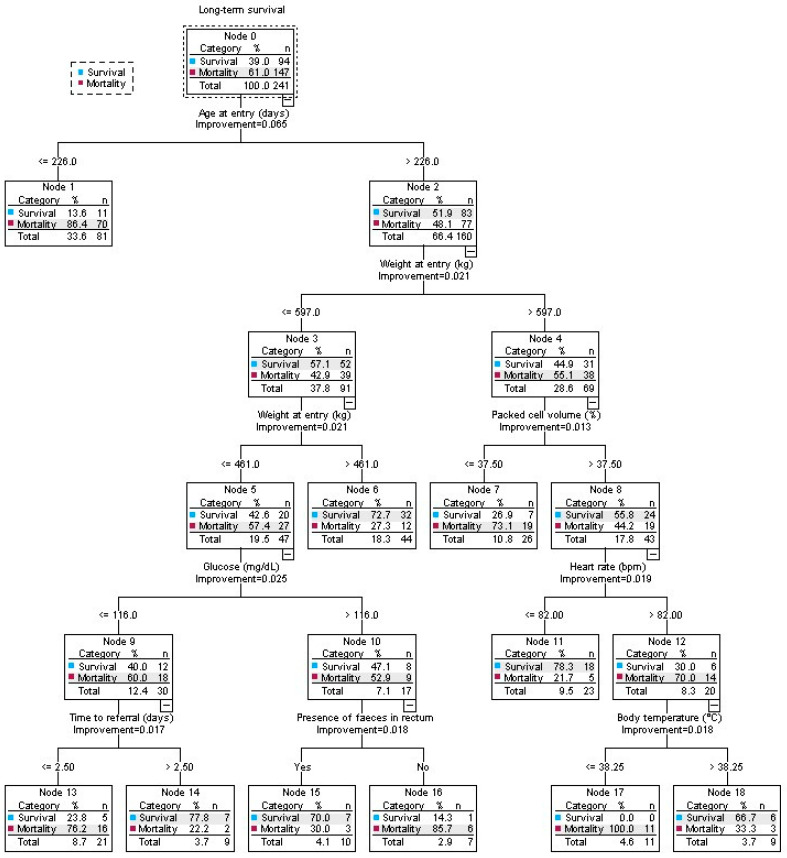
Explanatory decision tree for long-term survival of 241 cattle with intussusception based on clinical, ultrasonographical and laboratory findings available upon admission. Sensitivity = 74.5%; specificity = 83.0%; accuracy = 79.7% (Belgium). Abbreviations: *n*, animals included; bpm, beats per minute.

The output of the final two multivariable survival models with sensitivity, specificity and accuracy can be found in Table 5. The interaction between heart rate > 95 bpm and PCV was not significant (*p*-value = 0.38). Additionally, the interaction between male gender and time to referral > 4.5 days was not significant (*p*-value = 0.75). (Model 1: *p*-value = 0.96; Model 2: *p*-value = 0.53). In both models, the proportional hazard assumption was satisfied. In Figure 4, the final decision tree can be found, consisting of the following parameters: age at entry, weight, PCV, glucose concentration, heart rate, time to referral, presence of feces in rectum and rectal temperature. Sensitivity, specificity and accuracy of this tree were 74.5%, 83.0% and 79.7%, respectively.

## 4. Discussion

Despite intestinal intussusception being one of the leading causes of colic in cattle, research on prognosis was limited to only two studies [5,15]. The present study offers several new insights, enabling us not only to estimate short-term survival (discharge from the hospital), but also long-term survival, meaning survival until natural death/euthanasia or harvest (slaughter).

The first objective of this study was to determine short- and long-term survival. The short-term survival in this report was 44.8%, which is in line with a recent study (44.4%), but higher than reported in 1997 (35%) [5,15]. This number is likely depending on clinic-specific criteria determining the postoperative hospitalization period and subsequent discharge. The cattle population in this study predominantly comprises Belgian Blue cattle, the dominant beef breed in this country. This prevalence is driven by the breed’s elevated economic value, leading to a greater willingness to undertake more expensive surgeries. Therefore, Belgian Blue cattle, along with other high-value beef breeds, constitute a targeted population for surgical interventions. While Belgian Blue cattle may exhibit increased susceptibility to specific diseases, there is no evidence suggesting a divergence in survival rates for intestinal surgery. The survival rates observed in the current study align with those reported in previous studies involving other breeds [5,15]. Our study focused on data from cattle presented at a single university teaching hospital, in contrast to the study with a slightly lower prognosis, which incorporated medical records from cattle admitted to 17 veterinary medical teaching hospitals [5]. More advanced medical techniques might have contributed to this increase in prognosis (35 to 45%). Comparison with equine medicine revealed lower survival rates in cattle. Horses, benefiting from more sophisticated techniques and materials, showed survival rates for intussusception of 81–91% at hospital discharge and 48–68% one year post-surgery [20,21]. Survival in horses is affected by many factors, including the amount of contamination during surgery, surgeon experience, length of the intussusception, whether resection and anastomosis are needed, and the time to referral [20,21]. In this dataset, the maximum resected length of intestine was 150 cm, an important consideration for surgeons/practitioners when contemplating whether to continue surgery in cases of large affected pieces of intestines. In cattle, mortality primarily occurs during or shortly after surgery for intussusception. A first explanation for this might be a delayed referral by practitioners (average 2.3 days after first clinical signs). In addition, the more challenging detection of clinical signs in cattle by the animal caretaker could have contributed to this. Cattle, being prey animals, are stoic and quiet colic sufferers with vague clinical signs of intussusceptions, making them difficult to detect in a group of animals [22]. In addition, initial conservative therapy with spasmolytics further complicates matters as valuable time is wasted, potentially impacting the extent of necrotic bowel [12]. Subsequently, necrosis can result in leakage of intestinal fluid and thereby causing peritonitis [23]. Another explanation for the high mortality and more frequent euthanasia during surgery in cattle is the difficulty in locating the intussusception during clinical examination and ultrasonography, and the species’ anatomical limitations. For instance, the spiral colon is difficult to exteriorize [14]. Plus, the short, fat-laden mesentery complicates surgical procedures, making end-to-end anastomosis of the colon in a standing cow impossible [24]. It can only rarely be resolved by traction reduction, so euthanasia was often the outcome in the present study [23,25]. Surgery could be performed in a recumbent or anesthetized animal, but this requires more people and specialized facilities, thus a bigger investment of the owner. The latter, economic limitations of livestock owners are a final crucial argument for the higher mortality and euthanasia rates in cattle compared to hobby or companion animals. During hospitalization > 48 h after surgery, 32 animals died, likely as a consequence of strictures or intestinal leakage after resection and anastomoses leading to peritonitis. However, these deaths might also result from preoperative existing peritonitis non-responsive to therapy or other unrelated multimorbidity (e.g., pneumonia), although no specific information on the latter was available.

The second aspect was determining the long-term survival, which is what economically counts for the farmer as it provides the probability of an economic return (slaughter after a productive life). The overall long-term survival rate was 39.0%. Of the 108 discharged animals, 13% (14) died prematurely. Unfortunately, reasons for post-discharge mortality could not be retrieved, making it challenging to establish a direct link with intussusception or identify potential involvement of an entirely different disorder. However, seven survived for at least one additional year post-discharge before spontaneous death or euthanasia (four animals ≥ 3 years). In these cases, the likelihood of intussusception as the primary cause of death was unlikely. An encouraging finding is that the majority (87.0%) of animals discharged from the clinic were able to have a productive life, in a substantial number of cases even for several years. If we neglected all animals euthanized during surgery, the long-term survival rate rose to 55.9% (95/170). Presenting this information on prognosis to the farmers can aid them in making an informed decision regarding whether the cost of the performing surgery (e.g., resection and anastomoses) is worth it. Furthermore, analysis revealed a significant disparity in long-term survival between small intestinal (47.4%) and large intestinal (15.4%) intussusception (*p*-value = 0.002). Regarding the first objective, we can conclude that long-term survival was fair. However, more rapid referral could potentially reduce deaths within 48 h and thereby increase short- and long-term survival, since chances for further productive life post-discharge are high.

The second objective of this study was to determine predictive factors associated with mortality, which can potentially lead to a more accurate prognosis for long-term survival. It is important to note that specificity in our two multivariable models remained limited and therefore, other parameters should be considered when estimating a long-term prognosis. Model 1 included an increased heart rate as associated with a higher mortality, a parameter affiliated with stressful and painful situations such as acute abdominal pain [15,26]. A more extensive intussusception, involving a larger proportion of the mesentery, could lead to increased stimulation of nociceptive receptors and potentially a higher heart rate. In addition, extensive peritonitis due to leakage through necrotic tissue will also stimulate pain receptors. Previous research demonstrated that heart rate can be used to differentiate between healthy cows and those with traumatic reticuloperitonitis or abomasal ulcers and serve as a prognostic indicator in cattle with right-sided displacement of the abomasum and abomasal volvulus [27,28]. Similarly, in horses with colic, higher heart rates were associated with non-survivors [29]. While an increased heart rate can also be a sign of hemorrhagic or toxic shock, no significant association was found between these predictors. However, the higher physiological heart rate in calves compared to adults might be a confounding factor, since mortality is higher in calves. Next to heart rate, PCV was the second factor in Model 1, which showed an increase in mortality risk when <36%. Given that the physiological reference values for cattle fall within the range of 24–46%, PCV below 36% can be considered as normal [30,31]. When intussusception occurs, the animal likely experiences dehydration due to the impaired clinical condition and subsequent reduced water intake, along with the accompanying sequestration of fluid in the intestine. This can result in an increase in PCV. In cases with septic shock or toxemia, an elevated erythrocyte count can also be seen [32]. However, in the advanced stages of intussusception, bleeding may occur, manifesting itself as melena. This increased blood loss contributes to a decrease in the PCV, bringing it back to seemingly normal values and leading to higher mortality in these animals due to the advanced stage of intussusception. In the authors’ opinion, this model is easily applicable in the future for cattle suffering from intussusception. The variables incorporated in this model are objective and do not necessitate extensive laboratory analysis for determining their values, rendering them easily feasible for implementation in a clinical setting. However, it is important to note that the model was developed based on data from a second-line clinic, where animals often have a history of treatment and clinical signs over a period in time. Consequently, the application of this model in first-line veterinary practices may not be straightforward.

In Model 2, sex was a determining factor for mortality whereby male cattle had a significantly higher risk of not reaching slaughter. Male cattle are often reared extensively, contributing to a more difficult detection of the clinical signs by the animal caretaker. Once clinical signs are seen, however, the time to referral is similar in male and female cattle. Studies about bovine respiratory disease showed conflicting results for sex in relation to mortality [33]. However, it was demonstrated that the impact on mortality of respiratory disease, diarrhea, otitis and arthritis in veal calves, and of sepsis in critically ill calves, was significantly higher for male calves [34,35]. Interestingly, in horses with colic, the overall surgical survival rate in intact males is higher than females [36]. Notably, in our study, the mean and median age at entry was clearly lower for males compared to female cattle, which could have contributed to the higher mortality rates. A final factor in the second multivariable model was the time to referral. The biggest disadvantage for this parameter is the subjective nature. Clinical signs may be noticed earlier or later due to the farmer’s attentiveness, the latter being crucial for the (long-term) survival of the animal. However, in horses, time to referral is also detrimental to survival [21].

Decision-tree analysis highlighted additional factors of potential interest to predict survival in cattle with intussusception. Age at entry showed to be highly detrimental to the outcome, both in this decision tree as in the multivariable analysis as a container value. However, cattle aged > 226 days and weighing > 597 kg also displayed a higher mortality. A reason for this could be that in the authors’ setting, gastrointestinal surgery in heavier cattle is mainly conducted on standing animals using local anesthetics. Tension exerted on the mesentery during the surgical procedure triggers a pain response and can lead to the animal becoming recumbent [23]. This poses a risk of contamination of the surgical field and, in extreme cases, may result in unrepairable rupture of the intestines and mesentery. When farmers are highly motivated, surgery on an animal can be performed in lateral recumbency with general gas anesthesia. However, in these cases complications regarding regurgitation, respiratory problems, bloat and nerve damage also negatively affect the prognosis. Two other parameters that were included in the tree were glycemia and PCV, the latter already discussed above. Higher glucose levels give rise to a slightly better survival rate. Glucose is the main energy substrate during activities which contribute to acute emotional stress [37]. Transportation to the clinic and intestinal obstruction lead to higher glucose levels [38,39]. Lower glucose levels at arrival could indicate a longer reduced feed intake, associated with a less acute pathology. Lower concentration of glucose could also be due to the presence of bacteria which metabolize glucose in the peritoneal fluid, indicating septic peritonitis [40]. Other parameters included were heart rate, time to referral, the presence of feces and rectal temperature. Heart rate and time to referral were present in the multivariable Cox regression models as well, although cutoff values in the tree were slightly different. Counterintuitively, animals with a time to referral ≤2.5 days had a higher mortality than >2.5 days, but possibly the low number of observations in this node could have induced an age-dependent bias. Animals with feces in the rectum at entry had a higher survival rate than animals where no feces were present. When passage at the height of the intussusception is still possible, dilatation of the proximal intestines will be less distinct and impairment of the vascular system might have happened less extensively. Integrity and viability of the proximal intestines will be reduced alongside increased entrapment of fluids when a full obstruction occurs. An obstruction can also indicate a larger intussusception, whereby more necrotic tissue can be formed, leading to peritonitis. A final parameter, rectal temperature, showed higher mortality in animals ≤ 38.25 °C. Lower rectal temperatures can indicate the chronicity and severity of the pathology whereby shock, septicemia and hypovolemia are present.

The parameters derived from this study presuppose the presence of intussusception in the animals. Nonetheless, similar clinical signs may arise from other causes, rendering these parameters potentially less applicable. Nevertheless, with clinical examination, blood-gas analysis and ultrasound, a veterinarian can form a robust suspicion of intussusception, thereby aiding in the decision-making process between surgery and euthanasia using these predictive factors. A promising factor for further research is L-lactate. Lactatemia has been systematically measured in admitted animals since 2020, resulting in only 29 observations in this study. This limited power likely caused non-significant results for this parameter. However, the determined optimal cutoff value through ROC analysis was 6.4 mmol/L, comparable with that in another study in which a L-lactate concentration ≥ 6 mmol/L was shown to be associated with a negative outcome in Holstein dairy cattle with right displaced abomasum or abomasal volvulus [41]. L-lactate cow-side measurement might be a useful predictor of prognosis in cattle undergoing abdominal surgery. A larger dataset could allow determination of the actual relevance of this parameter as a predictor of survival in cattle with intussusception.

This study was subject to a few limitations: first, and most important, its retrospective study design. Over the 22-year study period, different veterinarians collected these data and an information bias could have occurred. Additionally, both inter- and intra-observer biases are possible as the description and interpretation of clinical findings can differ among veterinarians. Despite the long study period and diverse data collectors, efforts were made to maintain consistency in the pattern of examinations and data collection, aiming to mitigate this bias. A subsequent limitation arises from incomplete data for some animals. Confounding could have occurred if not all information was noted or recorded in the medical records, whereby other risk factors might have been present, but were not examined. There was no differentiation between mildly and severely dehydrated cattle as only normal (meaning skin turgor ≤ 2 s) and delayed (meaning skin turgor > 2 s) skin turgor were registered in the clinic’s system. This might explain why dehydration based solely on skin turgor was not a significant predictor. Additionally, multiple records lacked specific details on laparotomy, including information on the length of intussusception, its localization, vitality of the intestines and the presence of any leakage or peritonitis, which can provide valuable and crucial information on the survival of animals. Consequently, due to missing data, univariable and particularly multivariable analyses could only be performed with a restricted dataset. Incorporating the difference in prognosis between manual reduction and resection in this study would be valuable, as prognosis is likely better with manual reduction. However, due to limited cases where manual reduction could be performed, the power would be too limited to achieve any statistical effect; therefore, this analysis was not included. The inclusion of animals from various breeds and age also complicated the determination of optimal cutoff values, as physiological reference values for assessed clinical parameters differ between adult cattle and youngstock. Given that the dataset was exclusively derived from records of a single clinic, external validity may also be limited. All included animals were referred to a clinic, which required transportation, introducing time delays and subjecting the animals to additional stress. This could potentially influence clinical outcomes, resulting in a limited utility for practitioners. A last limitation involves the developed multivariable models and the decision tree. Due to the limited number of observations, the power was low and cross-validation was the only available validation method. No validation on new data was performed, limiting the accuracy of sensitivity and specificity estimation. As a result, the multivariable model and the decision tree should be regarded as purely explanatory and not predictive.

## 5. Conclusions

In conclusion, the present study showed the long-term survival of intussusception after surgical treatment to be fair in cattle. The mortality risk was highest during hospitalization in the first weeks following surgery. Factors of potential interest to estimate the mortality risk in individual animals were age and sex, which had a strong association with other factors, and heart rate, PCV and time to referral, as they appeared in both the multivariable model and the decision tree. Further exploration of other clinical factors or blood tests like L-lactate is needed to improve the ability to determine the long-term survival of intussusception. However, in cases with presumed intussusception, the information of the present study may already support the decision-making process of farmers and veterinarians to perform surgery or opt for humane euthanasia to the benefit of economics and animal welfare.

## Figures and Tables

**Table 1:** Descriptive results from blood-gas and electrolyte analysis at admission to the clinic in cases of intussusception in cattle (2001–2022, Belgium). Abbreviations: SD, standard deviation; min., minimum; max., maximum; pCO_2_, partial pressure of carbon dioxide.

Variable	Mean	Median	SD	Min.–Max.	Number of Cases (*n*)
pH	7.42	7.43	0.11	6.67–7.83	213
pCO_2_ (mmHg)	47.9	46.4	10.2	25.0–91.9	213
Base excess (mmol/L)	5.4	5.2	8.6	−30.8–34.2	228
HCO_3_^−^ (mmol/L)	30.0	29.4	8.1	5.5–60.3	212
Packed cell volume (%)	37.3	37.0	6.4	20.0–55.0	225
Sodium (mmol/L)	133.2	133.1	5.9	115.0–156.0	138
Potassium (mmol/L)	3.72	3.50	0.96	1.82–7.34	138
Ionized calcium (mmol/L)	1.01	1.01	0.12	0.71–1.28	143
Chloride (mmol/L)	87.6	89.5	10.1	65.0–105.0	98
Glucose (mg/dL)	111.3	107.0	48.4	28.0–331.0	95
L-lactate (mmol/L)	6.5	4.3	5.7	1.2–22.7	29

**Table 2:** Descriptives and results of the univariable survival analysis examining potential clinical and blood-based continuous predictors of mortality after surgical treatment of intussusception in cattle (2001–2022; Belgium). Abbreviations: SD, standard deviation; min., minimum; max., maximum; bpm, beats per minute; NSAID, non-steroidal anti-inflammatory drug; pCO_2_, partial pressure of carbon dioxide.

Variable	Cattle (*n*)	Mean ± SD; Median (Min.–Max.)		*p*-Value
		Slaughtered	Mortality *	
Age at admission (days)	241	871.3 ± 671.4; 680.5 (10.0–3016.0)	578.9 ± 729.4; 289.0 (4.0–4239.0)	0.004
Days of clinical signs (days)	208	±1.7; 2.0 (0.0–10.0)	2.5 ± 2.3; 2.0 (0.0–14.0)	0.11
Body weight (kg)	154	432.0 ± 218.2; 483.0 (50.0–890.0)	325.2 ± 258.1; 258.1 (42.0–800.0)	0.05
Heart rate (bpm)	235	81.2 ± 21.1; 80.0 (40.0–152.0)	93.6 ± 28.1; 93.0 (29.0–180.0)	0.001
Rectal temperature (°C)	236	38.5 ± 0.80; 38.6 (34.1–40.4)	38.6 ± 0.66; 38.7 (36.8–39.9)	0.20
Respiratory rate (breaths/min)	207	39.3 ± 15.0; 40.00 (12.0–80.0)	40.0 ± 17.5; 36.0 (12.0–88.0)	0.58
pH	213	7.43 ± 0.09; 7.44 (7.21–7.63)	7.41 ± 0.12; 7.43 (6.67–7.83)	0.26
pCO_2_ (mmHg)	213	46.6 ± 9.1; 44.9 (25.0–74.4)	48.7 ± 10.8; 47.1 (25.6–91.9)	0.26
Base excess (mmol/L)	228	6.1 ± 8.0; 5.3 (−12.3–34.2)	4.9 ± 8.9; 5.2 (−30.8–29.0)	0.41
HCO_3_^−^ (mmol/L)	212	30.4 ± 7.9; 29.2 (14.8–60.3)	29.7 ± 8.3; 29.5 (5.5–51.2)	0.61
Packed cell volume (%)	225	38.4 ± 5.4; 38.0 (27.0–49.0)	36.6 ± 7.0; 36.0 (20.0–55.0)	0.14
Sodium (mmol/L)	138	133.0 ± 4.8; 132.9 (120.0–142.0)	133.3 ± 6.6; 133.1 (115.0–156.0)	0.61
Potassium (mmol/L)	138	3.46 ± 0.72; 3.37 (1.82–5.23)	3.91 ± 1.07; 3.62 (1.90–7.34)	0.02
Ionized calcium (mmol/L)	143	1.01 ± 0.12; 1.02 (0.77–1.28)	1.01 ± 0.12; 1.01 (0.71–1.25)	0.74
Chloride (mmol/L)	98	87.7 ± 9.3; 89.0 (65.0–105.0)	87.5 ± 10.7; 90.0 (65.0–102.0)	0.72
Glucose (mg/dL)	95	118.2 ± 37.6; 114.0 (58.0–205.0)	106.3 ± 54.7; 103.0 (28.0–331.0)	0.44
L-lactate (mmol/L)	29	5.9 ± 5.2; 3.7 (1.5–20.4)	7.1 ± 6.2; 4.8 (1.2–22.7)	0.53
Intussusception length (cm)	63	62.0 ± 35.1; 50.0 (20.0–150.0)	60.1 ± 64.5; 40.0 (0.5–300.0)	0.85

* Stands for euthanasia and natural death.

**Table 3:** Descriptives and results of the univariable survival analysis examining potential clinical and blood-based categorical predictors of mortality after surgical treatment of intussusception in cattle (2001–2022, Belgium). Abbreviations: SD, standard deviation; min., minimum; max., maximum; bpm, beats per minute; NSAID, non-steroidal anti-inflammatory drug; SAID, steroidal anti-inflammatory drug; pCO_2_, partial pressure of carbon dioxide.

Variable	Categories	Observed Mortality	*p*-Value	HR	95% CI
Gender	Female (ref)	108/197 (54.8%)			
	Male	39/44 (88.6%)	<0.001	2.2	1.4–2.0
Breed	Belgian Blue (ref)	128/214 (59.8%)			
	Others	19/27 (70.4%)	0.30	1.3	0.80–2.1
Age at entry (days)	>226 (ref)	77/160 (48.1%)			
	<226	70/81 (86.4%)	<0.001	2.3	1.7–3.4
Season	Spring (ref)	40/74 (54.1%)	0.44		
	Summer	44/62 (71.0%)	0.10	1.5	0.93–2.2
	Fall	31/51 (60.8%)	0.52	1.3	0.73–1.9
	Winter	32/54 (59.3%)	0.59	1.1	0.71–1.8
Season hot/cold	Spring/summer (ref)	84/136 (61.8%)			
	Autumn/winter	63/105 (60.0%)	0.87	1.0	0.70–1.4
Weight (kg)	>305.5 (ref)	46/84 (45.1%)			
	<305.5	56/70 (54.9%)	0.007	1.5	1.2–2.5
Time to referral (days)	<4.5 (ref)	107/185 (57.8%)			
	>4.5	18/23 (78.3%)	0.04	1.3	1.0–2.8
Medication at home: SAID	No (ref)	124/211 (58.8%)			
	Yes	20/22 (90.9%)	0.006	1.8	1.2–3.1
Medication at home: spasmolytic	No (ref)	99/167 (59.3%)			
	Yes	45/66 (68.2%)	0.25	1.3	0.87–1.8
Medication at home: NSAID	No (ref)	99/156 (63.5%)			
	Yes (ref)	45/77 (58.4%)	0.63	1.0	0.64–1.3
Medication at home: antibiotic	No (ref)	95/163 (58.3%)			
	Yes	49/70 (70.0%)	0.12	1.3	0.93–1.9
Medication at home: NSAID+SAID	No (ref)	135/223 (60.5%)			
	Yes	9/10 (90.0%)	0.10	1.6	0.90–3.5
No medication at home	No (ref)	85/136 (62.5%)			
	Yes	59/97 (60.8%)	0.62	0.9	0.66–1.3
Colic	No (ref)	3/6 (50.0%)			
	Yes	54/98 (55.1%)	0.91	0.3	0.29–3.0
Temperature (°C)	>38.05 (ref)	115/182 (63.2%)			
	<38.05	28/54 (51.9%)	0.25	0.8	0.52–1.2
Heart rate (bpm)	<95 (ref)	72/144 (50.0%)			
	>95	70/91 (76.9%)	<0.001	1.8	1.4–2.6
Respiratory rate (breaths/minute)	<58 (ref)	103/175 (58.9%)			
	>58	23/32 (71.9%)	0.10	1.3	0.93–2.3
Pale mucosa	No (ref)	113/198 (57.1%)			
	Yes	28/33 (84.8%)	0.02	1.6	1.1–2.5
Capillary refill time (s)	≤2 (ref)	113/185 (61.1%)			
	>2	5/7 (71.4%)	0.61	1.4	0.51–3.1
Skin turgor (s)	≤2 (ref)	86/137 (62.8%)			
	>2	44/75 (58.7%)	0.78	1.0	0.66–1.4
Decubitus	No (ref)	132/216 (60.6%)			
	Yes	6/10 (60.0%)	1.0	1.3	0.44–2.3
Lumbar reflex	Positive (ref)	53/81 (65.4%)			
	Negative	29/52 (55.8%)	0.38	0.8	0.52–1.3
Tensed or bloated abdomen	No (ref)	21/39 (53.8%)			
	Yes	60/88 (68.2%)	0.05	1.7	0.99–2.8
Presence of feces in rectum	Yes (ref)	31/48 (64.6%)			
	No	79/128 (61.7%)	0.83	0.85	0.69–1.6
Melena present	No (ref)	1/5 (20.0%)			
	Yes	34/55 (61.8%)	0.21	1.3	0.49–26.3
Borborygmus present on auscultation	Yes (ref)	29/46 (63.0%)			
	No	77/133 (57.9%)	0.95	1.0	0.64–1.5
Rumen contractility on auscultation	Yes (ref)	4/10 (40.0%)			
	No	65/124 (52.4%)	0.59	0.86	0.48–3.6
Sloshing sounds on auscultation	No (ref)	44/70 (62.9%)			
	Yes	64/112 (57.1%)	0.46	0.83	0.59–1.3
Ping sounds on auscultation	No (ref)	54/98 (55.1%)			
	Yes	20/32 (62.5%)	0.67	1.0	0.66–1.9
Transrectal palpation: dilated intestinal loops	No (ref)	14/25 (56.0%)			
	Yes	50/105 (47.6%)	0.26	0.74	0.39–1.3
Transrectal palpation: donut-shaped structure	No (ref)	47/98 (48.0%)			
	Yes	17/32 (53.1%)	0.91	0.95	0.55–1.7
pH	<7.415 (ref)	59/89 (66.3%)			
	>7.415	68/124 (54.8%)	0.15	0.79	0.55–1.1
pCO_2_ (mmHg)	<44.95 (ref)	46/90 (51.1%)			
	>44.95	80/123 (65.0%)	0.15	1.3	0.91–1.9
Base excess (mmol/L)	<2.85(ref)	57/87 (65.5%)			
	>2.85	81/141 (57.4%)	0.33	1.0	0.60–1.2
HCO_3_^−^ (mmol/L)	>22.1 (ref)	103/181 (56.9%)			
	<22.1	23/31 (74.2%)	0.20	1.2	0.86–2.1
Packed cell volume (%)	<36.5 (ref)	76/108 (70.4%)			
	>36.5	59/117 (50.4%)	0.03	0.68	0.49–0.96
L-lactate (mmol/L)	<6.425 (ref)	8/19 (42.1%)			
	>6.425	7/10 (70.0%)	0.19	2.0	0.71–5.6
Sodium (mmol/L)	<139.70 (ref)	69/123 (56.1%)			
	>139.70	11/15 (73.3%)	0.26	1.2	0.76–2.7
Potassium (mmol/L)	<4645 (ref)	60/116 (51.7%)			
	>4645	20/22 (90.9%)	0.001	2.0	1.4–3.9
Ionized calcium (mmol/L)	>0.905 (ref)	69/119 (59.0%)			
	<0.905	13/26 (50.0%)	0.37	1.2	0.41–1.4
Chloride (mmol/L)	>89.50 (ref)	33/49 (67.3%)			
	<89.50	25/49 (51.0%)	0.25	0.62	0.44–1.2
Glucose (mg/dL)	<138.5 (ref)	50/78 (64.1%)			
	>138.5	5/17 (29.4%)	0.05	0.49	0.16–1.0
Ultrasound: Intestinal motility	Yes (ref)	44/66 (66.7%)			
	No	44/71 (62.0%)	0.70	0.88	0.71–1.6
Ultrasound: Intestinal dilatation	No (ref)	8/10 (80.0%)			
	Yes	127/214 (59.3%)	0.25	0.60	0.32–1.3
Ultrasound: Free fluid	No (ref)	24/36 (66.7%)			
	Yes	79/134 (59.0%)	0.46	0.78	0.53–1.3
Ultrasound: Donut-shaped structure	No (ref)	40/70 (57.1%)			
	Yes	28/52 (53.8%)	0.47	0.69	0.51–1.4
Location of intussusception	Small intestines (ref)	61/114 (53.5%)			
	Large intestines	33/39 (84.6%)	<0.001	2.0	1.4–3.4
Length of intussusception (cm)	<35 (ref)	15/22 (68.2%)			
	>35	18/41 (43.9%)	0.12	0.52	0.29–1.2
Peritonitis seen during surgery	No (ref)	12/24 (50.0%)			
	Yes	31/47 (66.0%)	0.28	1.1	0.74–2.8
Adhesion noticed during surgery	No (ref)	13/27 (48.1%)			
	Yes	24/36 (66.7%)	0.19	1.1	0.79–3.1
Postoperative antibiotics	Broad spectrum (ref)	43/104 (41.3%)			
	Small spectrum	5/20 (25.0%)	0.27	0.53	0.23–1.5
Postoperative flunixin vs. other NSAIDs	Flunixin (ref)	53/122			
	Other NSAID	9/18	0.53	1.3	0.62–2.5

**Table 4:** Survival time (days) and age at mortality (days) for cattle that underwent surgery for intussusception (2001–2022, Belgium). Abbreviations: SD, standard deviation; min., minimum; max., maximum.

Variable	Mean	Median	SD	Min.–Max.	Number of Cases (*n*)
**Survival time (days):**					
All cases	322.3	7.0	520.5	0–2797	241
Male	30.5	1.0	103.6	0–598	41
Female	354.4	10.0	544.9	0–2797	188
Slaughtered	718.0	579.0	543.2	98–2607	82
Deceased	61.3	0	297.1	0–2797	147
Dismissed and non-slaughtered	623.1	323.0	784.4	19–2797	14
Dismissed and still alive	815.9	663.0	473.4	381–1916	12
**Age of mortality (days):**					
All cases	1003.1	848.0	931.5	5–5121	229
Male	161.9	64.0	186.9	7–637	41
Female	1186.6	1090.5	928.1	5–5121	188
Slaughtered	1653.7	1630.5	679.3	260–3580	82
Deceased	640.2	324.0	853.6	5–5121	147
Dismissed and non-slaughtered	1476.3	1104.0	1400.4	54–5121	14
Dismissed and still alive	1246.9	1226.0	491.5	540–2162	12

**Table 5:** Results of the final multivariable survival model for factors associated with mortality in cattle with intussusception (2001–2022; Belgium). Abbreviations: HR, hazard ratio; CI, confidence interval; Se, sensitivity; Sp, specificity; Acc, accuracy.

	Variable	Mortality	HR	95% CI	*p*-Value	Se (%)	Sp (%)	Acc (%)
**Model 1**								
*n* = 223	Heart rate > 95 bpm	70/91 (76.9%)	2.0	1.4–2.9	<0.001	60.4	49.4	56.1
	PCV < 36.5%	75/108 (69.4%)	1.5	1.1–2.1	0.025			
**Model 2**								
*n* = 208	Male gender	39/44 (88.6%)	2.1	1.4–3.2	<0.001	88.0	65.6	74.5
	Time to referral > 4.5 d.	70/91 (76.9%)	1.8	1.1–2.9	0.026			

## Data Availability

The data presented in this study are available on request from the corresponding author upon reasonable request.
